# The role of community health workers and local leaders in reducing attrition among participant in the AIDS indicator survey and HIV incidence in a national cohort study in Rwanda

**DOI:** 10.1186/s12889-018-5243-x

**Published:** 2018-03-09

**Authors:** Mwumvaneza Mutagoma, Dieudonné Sebuhoro, Jean Pierre Nyemazi, Edward J. Mills, Jamie I. Forrest, Eric Remera, Augustin Murindabigwi, Mouhamed Semakula, Sabin Nsanzimana

**Affiliations:** 1 Rwanda Biomedical Center, Ministry of Health, P. O. Box 7162, Kigali, Rwanda; 2 School of Public Health, University of Rwanda, Kigali, Rwanda; 3 Ministry of Health, Kigali, Rwanda; 4 MTEK Sciences Ind., Vancouver, Canada; 5 Basel Clinical epidemiology and biostatistics institute, University Hospital, Basel, Switzerland; 6 Swiss Tropical and Public Health institute, University of Basel, Basel, Switzerland

**Keywords:** Community health workers, Attrition

## Abstract

**Background:**

Retention of participants in longitudinal prospective surveys can challenging for population health researchers. Community health workers (CHWs) may help reduce attrition.

**Methods:**

We used data came from a longitudinal prospective household-based survey targeting women and men in Rwanda, collected between June 2013 and December 2014. The sample was drawn from a population that included all residents of all 30 districts, 416 sectors, and 14,837 villages in Rwanda. The outcome measure was time to loss-to-follow-up. Follow up visits occurred at three, six and nine, and 12 months. A Cox proportional hazards model was constructed to identify factors independently associated with time to loss-to-follow-up.

**Results:**

Overall, 14,222 respondents consented to be interviewed at baseline. At the end of 12 months of follow up, 13,728 were revisited and consented to participate at 12 months of follow up. The overall attrition rate was 8.0%. A majority of those lost (54.3%) were less than 25 years of age, male (55.1%), not living in union (67.3%), had no education level or had primary education level (71.4%), or were in the highest wealth index (54.2%). Compared to illiterate, secondary education was negatively associated with attrition.

**Conclusion:**

The Rwanda AIDS indicator and HIV incidence survey recorded a very high retention of participants after 12 months. CHWs and local leaders played a major role to reduce attrition rate and identifying factors associated with loss-to-follow-up can help CHWs strengthen the quality of longitudinal survey data.

## Background

Population-based, longitudinal cohort studies are often time consuming and expensive due to the large financial and human resources requirements for close follow-up of participants [1]. Turnover of participants, leading to attrition, can occur during the period planned for the study. Retention and maintaining contacts with participants in a conventional longitudinal cohort study is challenging and may threaten the validity of findings from the study [1, 2]. In low- and middle-income countries, tracking of participants can be difficult due to a lack of systems and tools for tracking individuals [3]. Within the context of HIV epidemiology, loss-to-follow-up has been recognized as a major contributor to bias [4].

A systematic review of HIV cohort studies in sub-Saharan Africa, estimated an average of 22.6% attrition in longitudinal studies, although this varies widely across African countries [4]. In Kenya, attrition has been reported to be about 23%, 13% in Nigeria, 4% in Tanzania, and 3% in South Africa [5, 6]. Loss to follow-up has been attributed to employment stability, education, marital status, and in some countries war and political instability. Although retention is challenging in developing settings, CHWs could be integrated to improve retention.

In sub-Sahara African countries, CHWs have been successfully used in the era of antiretroviral therapy (ART) scale-up to provide basic health care through task-shifting, and help patients with ART adherence [7]. The World Health Organization (WHO) has published recommendations on the use of CHWs, recognizing their role in improving access of health services in the community [8]. In Rwanda, CHWs are selected in the community they are serving as outreach focal points and have updated information regarding the community within their jurisdiction. Although the benefits of CHWs for providing care services are well-documented, the aim of this study was to measure and report on the retention of participants in a nationwide cohort study, where CHWs were used in promote retention in the country’s 2013-2014 household survey.

## Methods

### Study design & Population

We utilized a population-based prospective cohort survey conducted from June 2013 to December 2014, using the previous Rwanda Demographic and Health Survey (2010 RDHS) sampling frame. A nationally representative sample of 12,792 households was selected. We applied a two-stage sampling design. Once a village was selected, local leaders organized a meeting, one week before data collection period, to explain the purpose of the study. Boundaries and household members in the selected village were identified by CHWs and local leaders helping in field data collection. A probability proportional to the village size was used in the first-stage and 492 villages were selected. We then conducted mapping, numbering and listing of all households in the selected villages. The list of all households in the village was considered as a sampling frame of the second stage. The target population was all women aged 15-49 and men aged 15-59. The purpose of the survey was to estimate syphilis and HIV prevalence in the general population and to estimate the HIV incidence after 12 months of follow up of HIV-negative participants. In all phases of the study, CHWs and local leaders were guiding data collectors to identify household members and calling them to schedule appointment in case of their absence at the data collection time. Wherever possible, we recorded the telephone number of participants in a register for subsequent follow up in the cohort study. One local leader per administrative unit (the village) facilitated the administrative organization of the survey. At least three CHWs per village were involved. Often, the local leader encountered in the village was either the chief leader of the village or the person in charge of security in the village. Villages’ local leaders and CHWs had an updated register of all inhabitants in the village, and most often were known to each other.

We recruited consenting eligible participants in the process of the study if resided in the household at the data collection start. Women aged 15-49 years old and men aged 15-59 years old signed the consent form. For participants less than 18 years old, informed parental consent was obtained from the parents or legal guardians of these participants. Follow-up visits occurred quarterly, with HIV testing occurring at baseline and 12 months of follow-up. The main purpose of these three quarterly follow up visits was to locate participants in the study, to know the number of those still committed to participate in the study in order to minimize the attrition rate during 12 months of the survey duration. The second objective was to strengthen the linkage of HIV-positive people to HIV treatment program in health facilities. HIV and syphilis positive participants were linked to care and treatment service in the nearest health facility. A schedule for taking laboratory results was communicated to participants through CHWs who acted as a link between health facilities and the community to sensitize and to remind participants to take their laboratory tests results. A five-day training was conducted for CHWs and local leaders to be informed about the duration of the study and issues of lost to follow up. Incentive was planned for them to be involved and committed to minimize the loss-to-follow-up.

### Study Measures

The primary outcome for this study was time to loss to follow-up, defined as attrition from the longitudinal study for any reason. Documented reasons, such as relocating or death, were also recorded. The household survey was primarily designed to assess the link of HIV testing to care and treatment and recorded demographic factors, as well as information on sexual behavior, knowledge and attitudes at each study visit. We collected data via Personal Data Assistant (PDA) devices and mobile phones to facilitate regular communication with study coordinators and participants and communicate with health facilities providing laboratory test results.

### Data Analysis

We applied descriptive statistics to characterize the distribution of participants in the study. Categorical data were presented using frequencies and percentages and continuous data by medians and interquartile ranges (IQR). A Cox-regression model was constructed to identify factors independently associated with loss to follow-up in the cohort. The outcome of interest in this model was the loss-to-follow-up of participants in the study, regardless of reasons, at each time of follow up assessment. The number of participants was different according to the time of follow up. Estimates are presented as crude hazard ratios (cHR) and adjusted hazard ratios (aHR), with associated 95% confidence intervals (95% CIs). All statistical tests were two-sided with alpha set at 0.05 Data were analyzed using STATA 12 software (StataCorp LP, 4905 Lakeway Drive, College Station, TX, USA).

## Results

**Fig. 1:**
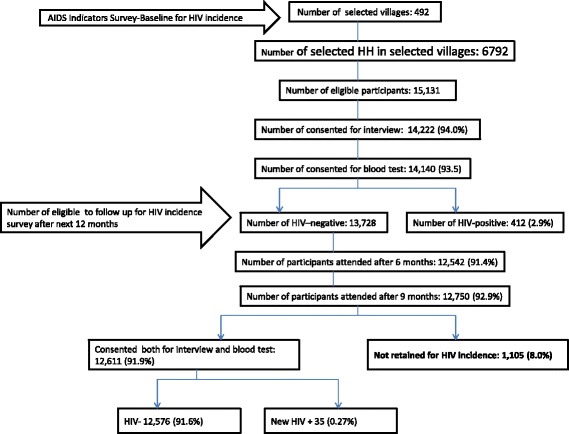
Rwanda AIDS indicators and HIV incidence participants’ recruitment flow

At baseline, within 6792 selected households, 28,938 household members were listed. Among them, 14,456 (50.0%) were eligible for the study. A total of 14,222 (98.4%) of eligible respondents consented to be interviewed and 14,140 (99.4%) consented for a blood test for HIV and syphilis. The baseline HIV prevalence ratio was 412/14,140 (2.9%). After six months, the number of participants retained in the survey was 12,542 (91.4%), 12,750 (92.9%) after nine months, and 12,611 (91.9%) after 12 months of follow up. Figure 1 displays the flow chart of study participants for the longitudinal cohort. At the end of the 12 months period of follow up, 35 (0.27%) people who were HIV-negative at baseline at sero-converted.

Table 1 displays characteristics of all participants and those not retained in the study either by refusal or unable to participate (including death) at the end of the study period. In total, 1,105 out of 13,728 (8.0%) did not participate in the end point phase of the study for any reason. The majority 600 (54.3%) of them were under 25 years of age and a majority were male 612 (55.4%). Of the participants who did not attend the end point study for any reason, 744 (67.3%) were not living in union, 789 (71.4%) had no education level or had primary education level and 699 (54.2%) were in the highest wealth index category. Thirteen (1.2%) were sick during the data collection period and 20 (1.8%) died during the follow up period.

**Table 1:** Characteristics of attrition in RAIHIs cohort study

	Reason for attrition (The denominator is total LTFU^a^)						Total (The denominator is the total sample size)	
	Unknown reason		Sickness		Died			
	n	%	n	%	n	%	N	%
Overall	1072	97.0	13	1.2	20	1.8	1105	8.0%
Age group								
16-24	592	98.7	4	0.7	4	0.7	600	4.4
25-49	457	96.2	9	1.9	9	1.9	475	3.5
50+	23	76.7	0	0.0	7	23.3	30	0.2
Sex								
Male	596	97.4	3	0.5	13	2.1	612	4.5
Female	476	96.6	10	2.0	7	1.4	493	3.6
Marital Status								
Not living in union	729	98.0	8	1.1	7	0.9	744	5.4
Married/Cohabiting	343	95.0	5	1.4	13	3.6	361	2.6
Residence								
Rural	764	96.5	12	1.5	16	2.0	792	5.8
Urban	308	98.4	1	0.3	4	1.3	313	2.3
Province								
North	121	95.3	3	2.4	3	2.4	127	0.9
South	179	95.7	2	1.1	6	3.2	187	1.4
East	230	97.5	2	0.8	4	1.7	236	1.7
West	197	94.7	6	2.9	5	2.4	208	1.5
City of Kigali	345	99.4	0	0.0	2	0.6	347	2.5
Religion								
Christians	1000	97.0	12	1.2	19	1.8	1031	7.5
Other	72	97.3	1	1.4	1	1.4	74	0.5
Completed level of education								
No education/primary	757	95.9	13	1.6	19	2.4	789	5.7
Secondary/vocational/high education	315	99.7	0	0.0	1	0.3	316	2.3
Wealth index								
Lowest/second/middle	481	95.1	12	2.4	13	2.6	506	3.7
Fourth/highest	591	98.7	1	0.2	7	1.2	599	4.4

^a^LTFU: Lost to follow up

Table 2 displays the results of the unadjusted and adjusted multivariable Cox-regression model. To model factors associated with loss-to-follow-up, the outcome was time to loss-to-follow-up for 1,072 out of 13,728 (7.8%), compared to those with longer (or complete) follow up time. Factors independently positively associated with time to loss-to-follow-up included being younger than 25 years old (aHR=1.7[95% CI:1.3-2.3]), 25-34 years aged (aHR=1.5[95% CI:1.2-2.0], being male (aHR=1.3[95%CI:1.2-2.5]), not living in union (aHR=1.5[1.1-2.0]), being single (aHR=1.8[95% CI: 1.6-2.2]) and being in urban residence (aHR=2.7[95% CI: 2.4-3.1]). Compared to illiterate, secondary education was negatively associated with attrition (aHR=0.8[95% CI:0.6-0.9].

**Table 2:** Attrition associated factors after 12 months of follow up in RAIHIS study

Characteristics	Bivariate model		Adjusted model	
	cHR	[95% CI]	aHR	[95% CI]
Age group				
15-24	2.6	[2.1-3.3]	1.7	[1.3-2.3]
25-34	1.7	[1.4-2.3]	1.5	[1.2-2.0]
35-44	1.4	[1.1-1.9]	1.5	[1.1-1.9]
45+	1.0		1.0	
Sex				
Female	1.0		1.0	
Male	1.3	[1.2-1.5]	1.3	[1.2-1.5]
Marital status				
Living in union	1.0		1.0	
Divorced/separated/widow	1.4	[1.0-1.8]	1.5	[1.1-2.0]
Single	2.3	[2.0-2.6]	1.8	[1.5-2.2]
Education				
Illiterate	1.0		1.0	
Primary	1.2	[1.0-1.4]	0.9	[0.7-1.0]
Secondary	1.6	[1.3-1.9]	0.8	[0.6-0.9]
Higher	2.9	[2.1-3.8]	1.2	[0.9-1.7]
Residence				
Rural	1.0		1.0	
Urban	3.0	[2.7-3.4]	2.7	[2.4-3.1]

## Discussion

Overall attrition in the 2013-2014 national household longitudinal survey in Rwanda was estimated at 8.0%. CHWs and local leaders played a considerable role in identifying eligible households and retaining them in the 12 months of follow up. The success of CHWs involvement in implementation of health services may be the result of continuous training and supervision by health professionals, and identifying factors associated with loss-to-follow-up in this study, including younger age, males, singles or not living in union and living in urban area, may help CHWs better retain participants in future longitudinal surveys.

The small size of Rwanda may be a facilitating factor in this low attrition rate. This is not only because transportation is easier, but also because community members often know where participants relocated to if they moved. The commitment of CHWs and local leaders also participated in national media and were also likely key factors in reducing the attrition rate. The willing of participants to know their HIV and syphilis status and to get treatment for free was a motivation to participate in the study. In another study conducted in Rwanda among adults on antiretroviral therapy (ART), where CHWs were also involved, although the population was quite different due to their vital interest to participate, the attrition rate was 9.6% at six months and 12.4% after 12 months [9]. This likely indicates that CHWs have become better at retaining participants in Rwandan surveys over time. Justman et al, in Swaziland, conducted a similar study and found an attrition rate of 6.0% after 6 months of follow up [10].

These findings compare similarly to a Tanzanian study (4.0%) and South African study (3.4%) where community or local leaders were involved [4, 5]. Kasirye et al found an attrition rate of 25% in Uganda using a household based four-year cohort study [11]. In South Africa, the attrition rate might vary by setting and population study. For instance in one study the refusal rate among attrition after 12 months of follow up was 1.2% was around 1% [5], in another study the lost to follow up was respectively 7.7% and 11.4% after six months [12].

Most participants lost-to-follow-up were young males. This is possibly explained as migration due to employment and/or education, is culturally more prevalent among men. Migration of young people between 15-24 years is estimated by the United Nations at 12.4% of all international migrants, mostly among young men [13]. A report produced conjointly by the Global Migration Group and the United Nations Children’s Fund stated that the main reasons of migration in African countries are related to employment, education and health [14]. In Kenya for example, the main reason for attrition in a household survey was due to employment needs [4]. Compared to illiterate, respondent with secondary education were likely associated with low rate of attrition. The assumption is that most of this category of people have telephone. In the follow up process telephones were used to track their new location. In Uganda, in 2001, graduated males were three times more likely to migrate than their counterpart graduated females [4]. The same situation was found in southern Tanzania where young single males were the biggest migrant group, and most went to Dar es Salaam for employment purpose [7]. In 2010, the African development bank group found that most migrants are educated and over 70% were males [15]. We also found that 67.3% of attrition cases in our study were not living in union. This is also common, as migrating would be much more constraining with the pressures of a family. Participants from urban setting were more likely to associated with attrition. The main reason is the unstable residence for participants who have not their own houses. Even in Uganda this issue was described where the attrition rate was double in urban setting compared to rural setting respectively 20.7% and 45,6% after four years of follow up [11].

Among the limitations in our study is the paucity of published data in the same domain and a control study for comparison. The study was also limited due to its longitudinal cohort design rather than randomized evaluation of CHWs versus some other attrition intervention. Some participants in the study did not have telephone or other contacts to locate them, others were sick, others died during the period planned for the study, thus unable to participate. Individual skills and experience of data collectors, limited data on demographic characteristics and limited time of follow up due to constrained resources might affect the quality of data, but due to the low rate of attrition, results from the study are accurate.

## Conclusion

A household-based cohort study success in the general population requires involvement of CHWs and local leaders’ collaboration. Capacity building strengthening of CHWs, their motivation and a tracking mechanism can reduce the lost to follow up of participants in this kind of study.

## Abbreviations

aHR: Adjusted hazard ratios
AIDS: Acquired immunodeficiency syndrome
ART: Antiretroviral therapy
cHR: Crude hazard ratios
CHWs: Community health workers
CIs: Confidence intervals
HIV: Human immunodeficiency virus
IQR: Interquartile ranges
PDA: Personal Data Assistant
RAIHIs: Rwanda AIDS indicator and HIV incidence
UN: United nations
WHO: World Health Organization
